# “Playing the Numbers Game”: Evidence-based Advocacy and the Technocratic Narrowing of the Safe Motherhood Initiative

**DOI:** 10.1111/maq.12072

**Published:** 2014-03-06

**Authors:** Katerini T Storeng, Dominique P Béhague

**Affiliations:** Center for Development and the Environment, University of Oslo London School of Hygiene and Tropical Medicine; Medicine Health and Society, Vanderbilt University, Department of Social Science, Health and Medicine King's College London London School of Hygiene and Tropical Medicine

**Keywords:** global health, evidence-based policy, audit culture, advocacy coalitions, maternal health

## Abstract

Based on an ethnography of the international Safe Motherhood Initiative (SMI), this article charts the rise of evidence-based advocacy (EBA), a term global-level maternal health advocates have used to indicate the use of scientific evidence to bolster the SMI's authority in the global health arena. EBA represents a shift in the SMI's priorities and tactics over the past two decades, from a call to promote poor women's health on the grounds of feminism and social justice (entailing broad-scale action) to the enumeration of much more narrowly defined practices to avert maternal deaths whose outcomes and cost effectiveness can be measured and evaluated. Though linked to the growth of an audit- and business-oriented ethos, we draw from anthropological theory of global forms to argue that EBA—or “playing the numbers game”—profoundly affects nearly every facet of evidence production, bringing about ambivalent reactions and a contested technocratic narrowing of the SMI's policy agenda.

We need new arguments. We have been saying the same thing for twenty years and it still doesn't resonate.

—Member of the Safe Motherhood Initiative's Secretariat (1987–2005)

## Introduction

In October 2007, nearly 2000 delegates from 115 countries gathered in London at the high-profile Women Deliver Conference to bring “new ammunition to the case for investing in maternal and newborn health” (Women Deliver 2007). Convened on the 20th anniversary of the 1987 UN-sponsored conference that launched the international Safe Motherhood Initiative (SMI) in Nairobi, Kenya (Starrs 1987), Women Deliver sought to reinvigorate global agencies’ and donors’ commitment to prioritize maternal health in low-income countries.

Between these two conferences, however, a major shift occurred in the types of arguments used to demand international action. In 1987, the call to action invoked a sense of international responsibility, underpinned by a strong feminist commitment to improving women's status. The initiative's founders accused the international community of neglecting the health of poor women in favor of a narrow focus on population control and child survival. Maternal mortality and morbidity in low-income countries (i.e., pregnancy-related death and illness) were said to remain high in part because of gender and economic inequities, or as argued in one editorial, because those affected by it “have the least power and influence in society” (Starrs 1987:4–5). Early safe motherhood champions, including the World Health Organization's (WHO) Director General Halfdan Mahler, claimed that reducing maternal mortality would demand not technical magical bullets, but comprehensive, multi-sectoral approaches to tackling the social determinants of maternal mortality, including women's low social status (Mahler 1987).

By contrast, those spearheading Women Deliver 20 years later advanced their claims through what conference organizers referred to as “evidence-based advocacy.” This term was used to highlight the importance of persuading the broader global health community to invest in maternal health not by making explicit moral claims, but by using quantitative objective evidence. In this sense, evidence-based advocacy is related to the broader shift in the 1980s and 1990s toward evidence-based medicine and evidence-based public health, both of which have contributed to the growth in monitoring of health targets in so-called developing countries (Greenhalgh 1996; Justice 1986) as well as the impetus to render health policy-making more objective, effective, and economical and less subjective and ideological (Dobrow et al. 2004). Indeed, the most valuable type of evidence that maternal health advocates now point to is the gold standard of cost-effectiveness evidence, used to calculate both the health and economic value of proposed interventions for reducing maternal mortality. It was based on this type of evidence that organizers of the Women Deliver conference devised as their slogan: “Invest in Women—It Pays.”

However, as a term, evidence-based advocacy (EBA) was rarely used in the 1990s, and as we began exploring its rapid emergence over the past decade, it became clear to us that EBA is a particularly vibrant emic notion currently undergoing significant flux. On one level, SMI actors’ adoption of EBA simply indicates their recognition that science holds considerable sway, particularly in the context of a proliferation of agencies, NGOs, and coalitions competing for funds and recognition on the global scene (McCoy et al. 2010). They may thus be demonstrating a certain pragmatism in relation to the rise of what Strathern (2000) has argued is the broader infiltration of an “audit culture” in various sectors with the rise of neoliberalism and the waning of trust in the authority of public sector institutions.

Certainly, many maternal health experts have become keenly aware that debates about the merits of scientific evidence in policy-making are often less about pure epistemic values than they are about the significant, recent expansion of a business-oriented ethos and audit-based orientation to global health (Birn 2009). Within this context, they have embraced EBA to bolster the SMI's marketable, credible, and scientifically authoritative identity, and to demonstrate its “value for money.” Yet, while advocacy experts within the SMI have become skilled at using EBA, they have also developed new EBA techniques with a sense of irony and considerable ambivalence, noting the way EBA undermines the potential of research to answer core questions relating to health systems, equity, and universal health rights, values to which they remain politically committed. Moreover, the term is increasingly used cynically to highlight that the use of evidence to “sell” safe motherhood to global donors sits in detrimental contradistinction to the more necessary problem-solving and analytical form of evidence-based policy-making.

Drawing on an ethnography of the SMI and anthropological theory of global forms, this article examines the contested rise of EBA. As we have noted elsewhere (Béhague and Storeng 2008; Storeng 2010), some maternal health experts respond to their sense of ambivalence regarding EBA by seeking to separate their contributions to advocacy from their core or “real research” projects. In this article, however, we demonstrate that the shift toward EBA is not merely discursive nor indeed easily contained in the world of advocacy. Instead, EBA—or “playing the numbers game,” as SMI actors often refer to it—has come to profoundly affect nearly every facet of evidence production, from how research becomes undertaken and evidence conceptualized, to how it is interpreted and presented, bringing about a greater degree of homogenization and technocratic narrowing in the SMI's policy agenda than its proponents had ever intended. We end the article by showing that it is in response to a growing sense of uneasiness with such technocratic narrowing that some experts are making new and creative uses of evidence in their efforts to reintroduce justice, equity, and rights into maternal health policy debates.

## The Safe Motherhood Initiative

The SMI was initially formed as an interagency group of UN actors (the WHO, the United Nation's Children Fund [UNICEF], the United Nation's Population Fund [UNDP], and the World Bank) that came together to raise donor commitment to maternal health in low-income countries. At the time, the specific term “safe motherhood” was coined to draw attention to how unsafe motherhood could be, but also because it was deemed an uncontroversial term, disassociated from ongoing debates in fertility control and abortion, yet encompassing a range of actions to improve women's health that would not antagonize socially conservative donors or governments (Storeng 2010).

Many of the SMI's founding members belong to the generation of women who participated in the antiwar and civil rights movements of the 1960s and 1970s and who worked in humanitarian roles in their early twenties, often as Peace Corps volunteers. Although most went on to train as doctors and statisticians, many retained their politicized interest in women's conditions, giving the SMI's early years its politically tinged dimension. Over time, however, the SMI has diversified and expanded to include a growing number of international and regional NGOs as well as other experts from multilateral and bilateral agencies, academia, and professional medical organizations. In 2005, for example, the Safe Motherhood Inter-Agency Group became formally incorporated into a new, WHO-hosted Partnership for Maternal, Newborn and Child Health (PMNCH).

Although a dedicated safe motherhood contingent remains, the overriding identity that typifies the SMI is bound less to its politicized origins than to the sociopolitical processes entailed in what political scientists refer to as “transnational advocacy networks” (Keck and Sikkink 1998) or “advocacy coalitions” (Sabatier and Jenkins-Smith 1993). A key *raison d’être* of such coalitions lies in competing with a growing and “unruly mélange” of other global health initiatives (Buse and Walt 1997:449). Many of these are public–private partnerships, the most prominent of which are the Global Fund to Fight HIV/AIDS, TB and Malaria and the GAVI Alliance. These have emerged with funding from business-oriented private sector actors—not least the Bill and Melinda Gates Foundation—and have challenged the position of the WHO as the undisputed leader in international health governance (Brown et al. 2006). Indeed, it is in part because of the growing sense of competition within the global health arena that, in the decades since the Nairobi conference, safe motherhood was strategically redefined (reluctantly by some) to refer to the more narrow and thus easier-to-advocate-for goal of reducing maternal mortality (women's death during or within 42 days of pregnancy) (Storeng 2010). Even so, the SMI has struggled to compete with more prominent global health initiatives (Shiffman 2007).

## Studying the Safe Motherhood Advocacy Coalition

After several decades of ethnographic research on the localized effects of globally derived health policies (Berry 2010; Castro and Singer 2004; Chapman 2003; Pigg 1997), anthropologists have begun to shift their attention to the emergence of a recognizable and powerful global health field (Adams et al. 2008; Biehl and Petryna 2013; Janes and Corbett 2009; Kapilashrami and McPake 2012). Janes and Corbett (2009) cogently argue that anthropological studies of global health policy should focus on the formation and dissemination of expert knowledge forms, in addition to their local consequences. Hardon (2005), for example, asserts that high-level global policy work often entails a focus on “magic bullets” that deny the complexity of local realities.

In dissecting the powers that enable these prescriptions, Nichter (2008:2) describes the role of global health elites and their use of scientific evidence to perpetuate “master narratives” that shape the very core of how solutions to global health problems are conceptualized, often in oversimplifying ways that assume universal applicability. Decoding such narratives necessarily entails attention to the social and political negotiations that go into their making, including the interrelationships between the various “substances of international health policy-making —[the] knowledge, ideology, politics of representation, competing vested interests, processes of persuasion and advocacy, etc.—[that] come to constitute it” (Janes and Corbett 2009:174).

Notwithstanding the strong homogenizing forces imbued within such master narratives, we contend that, taking from Ong's (2007:4) work on neoliberalism, advocacy coalitions are not systems as such, but are rather constituted by a “migratory set of practices … that participate in mutating configurations of possibility.” Thus, though coalitions are entrenched in broader global [health] forms, these global forms are not structural but have, as Collier and Ong (2005:11) assert, a “distinctive capacity for decontextualization, abstract-ability and movement … able to assimilate themselves to new environments [and] to code heterogeneous contexts and objects.” Though powerfully transportable, such global forms are nevertheless limited by specific technical infrastructures, administrative apparatuses, or value regimes (Collier and Ong 2005).

It is both the powerfully adaptive and limiting qualities of coalitions that we are interested in here—engendering “mutations” that we contend our informants are keenly aware of and make use of pragmatically, transforming evidence production in particular into a powerful tool for political, moral, and economic negotiations.

The ethnographic study of such dynamics entails a range of challenges. Not least of these is the reconceptualization of the ethnographic field as a social, political, and epistemic space that is diverse and that is articulated not in specific institutional or national settings but through geographically loose networks of relations and power (Shore and Wright 1997:14; Wedel and Feldman 2005). As Mosse (2011) has argued, studying global networks ethnographically requires a multifaceted approach for understanding the broader institutional contexts in which networks operate and through which specific perspectives, forms of knowledge, and practices are shaped and reproduced.

Our multifaceted approach included reviewing key international policy documents, scientific papers, and commentaries in major public health journals to identify trends in key policy debates. Between 2004 and 2009, we also conducted participant observation on safe motherhood research and policy networks while we were working as anthropologists within a number of interdisciplinary research projects on maternal health. In addition to day-to-day participation in research activities, we attended around 20 dedicated focusing events. These included research and advocacy meetings, high-level policy meetings and international global health conferences, including Women Deliver. At these meetings, we observed panel discussions and presentations and participated in informal discussions with a wide array of actors.

We also conducted formal, open-ended in-depth interviews with 72 individuals from the main organizations involved in the SMI. These included multilateral agencies, donor development agencies (U.S., U.K., and Norway), prominent research institutes, professional organizations for obstetricians and midwives, and international NGOs and private philanthropic foundations.

Because the safe motherhood community is among the smallest of the more influential global health coalitions, our ethnography has been particularly challenging to write about. To ensure our informants’ anonymity, we have at times had to sacrifice ethnographic details ideally needed to properly locate informants in time, place, and within a specific constellation of social relationships.

## Toward Evidence-based Advocacy

The drive for economic efficiency that is at the root of EBA preceded the formation of the SMI, starting almost immediately after the Alma-Ata Conference on Primary Healthcare in 1978. The conference embraced the goal of “Health for All by the Year 2000,” with primary health care being identified as a key means for achieving health as part of social and economic development (WHO and UNICEF 1978). However, within a political context of economic crisis and the ascendancy in major donor countries of a neoliberal ideology, a counter-movement pushing for *selective* primary health care quickly emerged. Selective primary health care focused on technical fixes to specific diseases that were deemed pragmatic, financially feasible, measurable, and politically unthreatening alternatives to the idealistic comprehensive primary health care agenda (Brown et al. 2006:67).

In its early years, the SMI was formed in part through an explicit rejection of the ethos of selective primary health care (Storeng 2010). Even so, the initiative quickly became bound up in a global health logic focused on the increasingly twinned issues of measurement and economic efficiency. Indeed, in a landmark article published in 1992, leaders in the field argued that the neglect of maternal health in resource allocation on the one hand, and the poor quality and scope of maternal health-related data on the other are “self-reinforcing and constitute a measurement trap” (Graham and Campbell 1992:967). This measurement trap, they claimed, had constrained efforts to establish the levels and trends of specific maternal health outcomes, to identify the characteristics and determinants of health outcomes, and to monitor and evaluate the effectiveness of programs.

The measurement trap problem became accentuated with the growing popularity of “burden of disease” priority-setting tools throughout the 1990s. Spearheaded by the World Bank, these tools cemented the idea that diseases and conditions accounting for a high burden of mortality and morbidity should be prioritized. Although maternal mortality had been shown to be the leading cause of death among reproductive-age women in low-income countries, the burden of disease logic fostered the key idea among global donors that the number of maternal deaths was too small relative to deaths from other global health problems like infectious diseases to warrant prioritization—an idea that maternal health advocates claim persists today. As a communication specialist from the D.C.-based Population Reference Bureau explained: “The fact is you really have a struggle because if you compare the number of deaths, there are half a million maternal deaths compared to 10 million infant and child deaths per year … people say it's nothing compared to some of the other issues.”

The establishment of the UN Millennium Development Goals (MDGs) in 2000, one of which is to improve maternal health, reinforced this demand for quantitative health indicators. As one informant explained, in the past “donors never wanted indicators and then they wanted results and everybody started asking ‘What are you using your money for?’”

At the same time, donors’ and policy-makers’ growing emphasis on experimental randomized controlled trials (RCTs) and cost-effectiveness calculations (the ratio of costs and health gain, such as years of life or disability-adjusted life years saved) posed significant problems to maternal health specialists, who were unable to produce convincing (experimental) evidence that the interventions recommended by the SMI are cost effective. This is because it is both expensive and impractical to conduct RCTs with maternal mortality as an outcome due to the very large sample size that is needed to be able to attribute differences in maternal mortality to a given intervention (Campbell et al. 1995). Above and beyond this, the recommended maternal health interventions—such as skilled birth attendance, emergency obstetric care, and good referral mechanisms—are complex and span different levels of the health system, and cannot easily be subjected to experimental study (Campbell et al. 1995). By the time we started our fieldwork in 2004, the dearth of RCT-based evidence on interventions to reduce maternal mortality had significantly reinforced the sorts of anxieties articulated in the 1992 measurement trap article (Béhague and Storeng 2008). According to one senior European maternal health epidemiologist, lack of experimental evidence had become a main impediment to the SMI's ability to compete with other global health coalitions that, as he put it, have “a better record of [providing] evidence-based recommendations.”

## Consolidating Evidence-based Advocacy

Evidence-based advocacy is much more effective than any other kind of advocacy that can just be written off as ideological.

—Senior international NGO representative

Certainly, all global health coalitions have responded to the drive for evidence-based efficiency and for the removal of ideological and moral arguments from the policy-making process. However, the SMI's original appeal to feminist and social justice arguments has added an additional burden and, according to some, become a veritable liability (Storeng and Béhague 2013). “I think everybody's afraid of getting the feminist label because it turns so many people off,” explained one researcher from a New York–based women's health NGO.

In fact, some informants had come to feel that because the original feminist ideological basis of the movement has been discredited, scientific evidence currently plays a particularly important role in compensating for the low political appeal of maternal health. In the early 2000s, key actors thus began to argue that the SMI would not achieve policy leverage and inclusion in high-level policy forums if it did not learn to talk more authoritatively through use of scientific evidence and health statistics.

It was during this explicit rearticulation of the SMI's identity away from its original political positioning that a growing array of EBA practices, to be described below, came to the fore. The main proponents of such practices have been advocacy and communication specialists based in international NGOs, which are strategically positioned in cities like Washington D.C., New York, and London to, in the words of one such actor, “influence the ‘high politics’ of global health.” U.S.-based NGOs have been especially fervent promoters of EBA, perhaps because they themselves have come under intense pressure from their funders (which often includes USAID and sometimes private donors like the Bill and Melinda Gates Foundation) to demonstrate their own value for money.

Although some of these NGOs have been active since the SMI's start, many are new entities sustained by the growing emphasis on “getting research into policy” and in particular, “knowledge translation,” a process whereby peer-reviewed scientific publications are simplified and made intelligible to busy policy-makers (Greenhalgh and Wieringa 2011). These new actors distinguish themselves from academic scientists; although they often conduct research as part of their work and recognize scientific expertise as key to their own political influence, they distance themselves from the publish or perish culture they see as constraining their academic counterparts. Nevertheless, NGO-based advocacy specialists are often aided by colleagues within UN agencies and by academics who have gradually, if reluctantly, embraced an advocacy role.

### Performance Indicators

Although statistical evidence was important to the SMI's initial success in drawing international attention to the issue of maternal mortality in low-income countries, the demand for numbers to set agendas has increased significantly with the emphasis on evidence-based policy-making. With time, advocacy specialists have become acutely aware of the political power of numbers, including the way in which numbers can help generate a political response where rhetoric alone seems ineffective. League tables ranking different countries’ maternal mortality ratios (MMRs) have proven to be particularly effective advocacy tools for generating political responses. A Belgian epidemiologist who contributed to the production of such a table in the late 1990s recalled how its publication sparked a major political response in one West African country that was ranked as having a higher MMR than its neighbor. “For the politician, [the league table] is all about how he is [to be] judged. Because unconsciously people concentrate on the numbers,” our informant explained, adding that the MDGs’ emphasis on measurable results has reinforced this tendency dramatically. For him, the evocative power the MMR indicator has acquired reflects that it has become an indicator not just of women's health, but of a country's overall development:

Maternal mortality has become sufficiently part of the collective conscience, so much so that is has become one of the Millennium [Development] Goals. It is now understood by policy-makers to be an important indicator of the performance of the health system, which, in turn, indicates the social performance of a country.

A high MMR thus creates an incentive for a country to prioritize maternal health he explained, if only to avoid appearing less developed than its neighbors.

The political power of numbers exists even when the validity of the numbers is contested. In fact, controversy over the validity of statistics can actually help galvanize a political reaction. Another epidemiologist at a leading American public health school had witnessed such a situation when the UN published a maternal mortality estimate for Morocco that was higher than the government's own census-based figure. The discrepancy between the numbers generated intense political debate about the government's accountability, trustworthiness, and commitment to women's health. As she recalled, “The opposition took this up and said, ‘Thank God there are international agencies who will tell us the truth about our women who are dying, the government is clearly lying to us.’ And this issue was debated in parliament … it certainly caused a big [political] brouhaha.” This reaction, she explained, occurred despite the fact that, at the technical level, the figures were not comparable because they had been derived using different methods.

Advocacy groups explicitly capitalize on political effects such as these. Several U.S. NGOs, for example, showed us the visual graphics and colorful “report cards” that they use in their yearly reports to compare countries’ performance on maternal health indicators. More prototypically, even, the Countdown to 2015 initiative established in 2005, in which a number of our informants were involved, has as its stated aim: “to monitor and hold countries accountable” for their progress toward the MDGs through collation and communication of statistics on the “coverage of health interventions proven to reduce maternal, newborn and child mortality” (Countdown to 2015 2009). The explicit premise of Countdown to 2015 is that drawing attention to performance indicators will stimulate “better and stronger efforts at the country level” by governments concerned with their image and keen to demonstrate the results of their donors’ investments (Countdown to 2015 2009).

### Creative Epidemiology

Advocacy specialists have also become adept at using what some public health specialists explicitly term “creative epidemiology” to underscore the attention-seeking aspect of research (Wallack et al. 1993). Displaying an acute appreciation for how distinct classes of stakeholders interpret numbers differently, several of our informants explained that the presentation of statistical data needs to be modified depending on the audience. For instance, different expressions of the statistical risk of maternal death should be used to influence global-level policy elites, national-level decision-makers, and the public respectively.

Although the MMR can be highly effective in influencing national-level policy debates, precisely because it is the key indicator that has been used to compare countries across the globe, the MMR was deemed too technical for the lay public. Rather, statistical expressions that are “more immediate, individual” and amenable, even, to be personalized—such as the lifetime risk of maternal mortality—were said to be easier for lay people to draw meaning from and thus more helpful in gaining the public's support for maternal health investment. As a senior advisor with the NGO who long served as the SMI's secretariat observed, “When you talk with people one to one and you say that one out of every six women in Afghanistan die in pregnancy and childbirth compared to one in every 30,000 in Sweden or Norway, people are absolutely horrified, shocked.”

Persuading global-level policy actors, in turn, demands different statistics, primarily because of the view that the number of maternal deaths is too low to warrant prioritization when compared with other health issues. As the senior advisor cited above explained, when it comes to a global health audience, “you really do have to frame it in a different way because the numbers [of maternal deaths] alone don't make the case.” One strategy has been to focus not only on mortality but also on producing new evidence of the maternal morbidities (and even long-term disabilities) that result from pregnancy-related problems in low-income countries (e.g., WHO 2004). Using these data, advocacy specialists are effectively able to argue that maternal mortality is only “the tip of the iceberg” of the neglect of pregnancy-related health.

Premised on the close clinical links between mothers, children, and newborns, expressing advocacy messages in terms of the combined burden of disease affecting these three groups has emerged as a creative strategy for making the problem of maternal mortality appear bigger and, by extension, more important. Unlike maternal mortality alone, one of our informants explained, when combined, the “MNCH” (maternal, newborn and child health) burden of disease far exceeds that attributed to HIV/AIDS, TB, and malaria, thus inflating the number of lives that can be saved by investing in maternal health and “making a much stronger advocacy argument.” Such anticipated benefits were one of the main reasons that many safe motherhood advocates pragmatically supported the SMI's involvement in the PMNCH, despite widespread concerns about undermining the SMI's longstanding effort to ensure safe motherhood not be overshadowed by the more philanthropically appealing issue of child health. As one senior U.K.-based maternal health researcher and advocate explained, “it makes sense to bung in [include] the babies for the numbers game.”

### Calculating Economic Impact

Deviating even further from the original SMI's political arguments, EBA has also entailed producing and using economic evidence to appeal to the dominant logic of cost effectiveness. A senior UNFPA adviser who was involved in elaborating the Women Deliver advocacy strategy insisted that “economic rebranding” has become necessary to “sell” safe motherhood. In her everyday work, she avidly endorsed activities that would be able to, as she put it, “position this product as an opportunity of desire so people will want to invest in it.” A founding member of the Safe Motherhood Inter-Agency Group shared this perspective: “You can mobilize a certain constituency group just by talking about the ethical and injustice issues, but for these hardcore decision-makers who look at economic factors, that kind of appeal doesn't necessarily carry the day.”

For many of our informants, then, a key way of ensuring that investing in maternal health is seen as a good “global health buy” (in the way, say, immunization or antiretroviral HIV medication are) is through the production of more experimental (RCT-based) evidence of cost effectiveness (Béhague and Storeng 2008). As noted above, however, this is almost impossible for complex health system approaches designed to reduce maternal mortality.

Rather than contest the epistemological limitations of the experimental method for demonstrating the impact of health systems strengthening and intersectoral innovation, as some academics have begun to do (Béhague and Storeng 2013), advocacy specialists have pushed for producing cost-effectiveness evidence of simple and targeted interventions, such as drug-based treatments for hemorrhage and infection (among the main clinical causes of maternal mortality). As one such informant put it, “donors want to show that they save lives. That's not what you get by putting your money into strengthening the health system or training a bunch of midwives.” Targeted interventions, another advocacy specialist implied, are pseudo-commodities that are easier to sell because their impact on deaths averted can be more easily demonstrated through gold standard experimental research. Advocates called for the production on evidence of such interventions to enable them to show that cost effective interventions for maternal health exist and that saving women's lives makes good economic sense. This is needed to achieve what several informants labeled “buy-in” from donors who are not easily swayed by calls for broader health systems development.

Advocacy specialists have also started to invoke broad macro-economic arguments about the importance of maternal health for economic development. “Women deliver so much more than babies,” the head of the Women Deliver advocacy team repeated on more than one occasion, explaining that the double meaning in the term “deliver” highlights women's combined reproductive and productive (economic) contributions to households and society. Inspired by the mobilization of similar arguments in the HIV/AIDS subfield, the Women Deliver conference became a showcase for new economic estimates of productivity loss and impoverishment resulting from pregnancy-related mortality and morbidity (e.g., Gill et al. 2007). Moreover, around the time of the conference, donors assessing cost effectiveness were urged to consider the full benefit of investing in maternal health, including not only women's survival and associated economic outcomes, but also the “knock-on” benefits for children's and newborns’ survival and the associated long-term benefits for national economic productivity and growth (e.g, The Lancet 2007).

So central have these kinds of EBA practices become that many advocacy specialists’ professional activities are now focused on “capacity building,” or teaching counterparts in low-income countries how to engage in EBA, whether by producing evidence themselves or, more commonly, learning how to implement knowledge translation programs in their own settings. According to one communication specialist, such capacity building can involve “flooding” local policy champions with evidence through workshops and training sessions to improve their bargaining position vis-à-vis their own governments and, especially, foreign donors: “I say [to them], ‘these are the talking points if you're going to go to USAID or if you're going to a safe motherhood meeting … this is why [this evidence is] relevant to your country.’”

## A Reluctant (and Critical) Evidence-based Advocacy

On many levels, the EBA practices described above have been highly successful in responding to the key structural and ideological changes in global health governance, namely increased competition for resources and a focus on quick, visible productivity and measurable accountability. There is no doubt that EBA has contributed to a recent surge in donor interest in maternal health, exemplified by the growth of donor commitment to large policy initiatives such as the Every Woman Every Child global movement launched by the UN secretary-general in 2010 and the Global Strategy for Women's and Children's Health (PMNCH 2011).

Advocacy experts’ success in engaging with this culture of objectivity, however, has been mixed in with a great deal of concurrent apprehension and intensely critical—and self-critical—views of the evidence-based activities in which many are partaking. According to several of our interviewees, the claim that safe motherhood recommendations are based on a particularly weak evidentiary base is unfounded and relates, rather, to the fact that donors’ and health policy-makers’ demand for scientific evidence is disproportionately high when it comes to maternal health. When asked about the reasons for this, one advocacy specialist pointed to a widespread lack of political commitment to women's health and disregard for a coalition that has traditionally been seen as being led by women and aligned to the women's movement. As she explained, HIV, for instance, captured attention not because it had good quality evidence, but because: “The first [donors and leaders] who got behind [HIV/AIDS] were males in the US! I mean, what stronger voices … can there be? And this [safe motherhood] is a story about mothers and children …. if we women talk about it, we're whining about a topic that is, you know, not interesting.” And as another advocacy specialist similarly highlighted: “How many billions of dollars have gone to abstinence programs for HIV/AIDS in the last six years? What's the evidence there? You can't take a fraction of that money to save a woman's life unless you have the evidence.”

Though critical views such as these were frequently linked to the underlying feminist concern that women's health is simply not a priority in what is still a male-dominated world, such insights were not gratuitous, but, rather, translated also into self-critical views and deep ambivalence regarding the benefits of EBA. This ambivalence reflected the ways it can easily do a disservice to the political commitment to equity and intrinsic health rights, while also contributing to a technocratic narrowing of the policy agenda.

### Technocratic Narrowing

In interviews, advocacy specialists reflected critically on the way their activities have contributed to narrowing the terms of debate about maternal health by restricting the kinds of arguments and forms of evidence that “count” as authoritative. “It's all quantitative now,” a communication specialist working for a D.C.-based international NGO commented. The president of a well-known advocacy and research NGO claimed that the perceived pressure to use evidence in policy debates has become so intense that whereas two decades ago key SMI leaders were able to write credible editorials centered on the importance of women's status and their right to health (e.g., Sai 1987), today they are no longer comfortable making policy statements based on such principled ideals if unsupported by scientific evidence.

Moreover, the idea that burden of disease and instrumental economic arguments should guide the allocation of resources is in irresolvable tension with many SMI actors’ private conviction that intrinsic commitment to social justice, rights and equity should be the main policy drivers. Many worried, for example, about endorsing priority-setting tools such as burden of disease analyses that have been shown to systematically bias priority away from conditions suffered by poor women, including pregnancy-related mortality and morbidity (e.g., Sundby 1999).

Several of those who had participated in the Countdown to 2015 and similar accountability projects demonstrated unsettled ambivalence about the fact that they had contributed not just to agenda-setting but also to the exportation of “target culture” to donor-dependent countries. They noted, for example, that the enforcement of accountability demands can encourage donor recipients to produce fake numbers. More importantly, responding to donors’ demands for numbers diverts attention from building sustainable and locally relevant health information systems. One longstanding safe motherhood advocate now working as a researcher with a Gates-funded NGO even lamented that her involvement in a project to promote a drug that can prevent maternal deaths from postpartum hemorrhage had probably contributed to narrowing the maternal health agenda from social and political solutions toward purely technical ones:

I'm actually doing something that philosophically I wouldn't have believed I would have done a few years ago because I do believe in holistic care and I do believe we need to … [tackle] the system. So what am I doing sitting here working on … I wouldn't say it's a silver bullet for maternal health … but, you know, one of the few things that comes as close to a kind of silver bullet strategy as you might get?

As we have described in greater detail elsewhere (Béhague and Storeng 2008, 2013; Storeng 2010), many maternal health specialists were also highly critical of the ways the demands for cost-effectiveness data reinforce technocratic narrowing by shifting research priorities away from the use of multimethod studies on the dynamics of maternal mortality declines—or, indeed, disaggregated local-level statistics and process evaluations of actual policy change—to a focus on targeted interventions that can more easily be subjected to experimental study.

### Evidence-based Politics

The kinds of critical insights we have described above are thus not simply rhetorical, but are, rather, beginning to translate into research practices of all kinds, including those being developed and supported by an increasingly prominent network of academics working alongside human rights lawyers. Though not radically different, and though still functioning within the basic parameters of EBA, these practices are being actively used to reintroduce justice, equity, and rights into policy-making, but on more “legitimate” scientific grounds.

Perhaps the most notable of these practices entails a return to epidemiology's historic focus on the social determinants of health, specifically, the use of disaggregated indicators to illustrate inequities in the socioeconomic, ethnic, and geographic distributions of maternal mortality, including disparities in access to life-saving services both between and within countries (Freedman 2003). This approach, as one lawyer and advocacy specialist explained, allows one to talk about inequities in an objective descriptive manner and, importantly, avoids using “off-putting rights language.” At the same time, within the context of a private exchange, she did not hesitate to interpret such disparities in political terms:

If you look at a country where you have a middle class that's able to give birth safely but then you see very high rates of maternal mortality among minority groups, immigrant groups, then it is clear you have a discrimination issue and that's not a difficult rights argument to make.

As she further explained, advocates using such data are beginning to effectively engage governments in a discussion about their accountability to the MDGs and, importantly, to international human rights treaties that, in principle at least, oblige states to address systemic discrimination and inequity through targeted policy action.

The kind of ideological pragmatism demonstrated here was also apparent in other advocacy specialists’ sophisticated involvement in the process of defining the target indicators to be used in global-level monitoring of countries’ progress toward the MDGs. SMI leaders and NGOs were, on the whole, excluded from high-level UN-based formulation of the Millennium Declaration in 2000 and the subsequent articulation of the eight MDGs. Though improving maternal health ended up being one of the goals (MDG 5), many felt that the international community, in focusing the goal exclusively on the target of maternal mortality reduction, had reneged on promises to promote the broader concept of sexual and reproductive health that had been made at the UN International Conference on Population and Development in 1994.

According to our informants, maternal health was chosen as the focus of MDG 5 because it is less controversial than reproductive health. However, to argue for an additional MDG on reproductive health once the goals had been set was deemed politically unwise. Instead, key individuals began to demand NGO representation on the expert panel drawn up to define targets and indicators for measuring progress towards MGD 5. Through this forum, they successfully argued that the existing *target* on maternal mortality reduction was too narrow to capture the broader *goal* of improving maternal health. In 2006, an additional target indicator—universal access to reproductive health—was finally included under MDG 5, the only negotiated MDG target to date. Though clearly only a partial victory, many within the field considered this an important achievement, reflecting their conviction in the often repeated maxim, “in public health, what you measure is what you do.”

## Conclusion

EBA has undoubtedly been successful. Within a context of growing emphasis on quantitative evidence to inform priority-setting and justify investments, playing the numbers game has emerged as perhaps the most viable strategy for global health initiatives like the SMI as they struggle to define their identity and compete effectively in a rapidly changing global health field. Indeed, EBA and its success is exemplary of the broader “audit culture” described by Strathern (2000) and others (Power 1997). As Shore and Wright (1999) claim, the expansion of audit tools from financial accountancy into other sectors such as education (and, we would argue, health) has enabled the expansion of neoliberal forms of governance, where professional relations are reduced to quantifiable and, above all, inspectable templates.

Indeed, such governmentalizing tendencies demonstrate the broader historical growth during the 20th century of the power of scientific authority, of a pervasive “trust in numbers” (Porter 1995) and culture of objectivity that has come to characterize modern societies (Daston 1992; Nader 1996). However, the rise of EBA highlights that cost-effectiveness evidence—the gold standard within evidence-based policy-making—is, in fact, not removed from ideology. Rather, it is itself another ideology that is being introduced and, as we have documented in this article, adopted and adapted with various degrees of ambivalence. Safe motherhood advocacy specialists recognize, as Haraway (1988) has pointed out, that claims for action gain more authority and legitimacy if they are dissociated from subjective ideologies and linked to high-status social agents, such as scientific evidence. One could even argue that advocacy specialists rely explicitly on the way in which health statistics acquire an authoritative social life of their own, despite the fact that they are also aware of the ways numbers can so easily be fraught with technical, and indeed ideological, difficulties (Hacking 2007; Nichter and Kendall 1991).

Our analyses support key findings from the anthropological study of global forms of expertise (Adams 2010; Lakoff 2010; Pfeiffer and Nichter 2008). We have, for example, underscored the way in which global health policy, and especially its emphasis on quantitative evidence, reinforces an oversimplified “master-narrative” circumscribed by technical solutions to health problems (Nichter 2008). As a result, moral and social justice arguments have become partially eclipsed by the more competitive uses to which evidence-based policy-making activities have been put.

Importantly, however, we have also highlighted the sociopolitical struggles and rifts that go into the making of these narratives, specifically the ways safe motherhood experts not only disseminate authoritative knowledge and create master-narratives, but also resist and modify them, showing both reflexivity and decidedly ambivalent attitudes to their own contributions to EBA (Béhague and Storeng 2013). In some instances, SMI experts cogently echo the kinds of criticisms that anthropologists often make regarding the growing marginalization of plural forms of evidence (Adams 2005; Biehl and Petryna 2013; Lambert 2006, 2013).

The alternative evidence-based activities some experts are engaging in (e.g., to keep a broad conceptualization of the social determinants of maternal health and rights on the agenda) give lie to the idea that ideology has been fully banished from the evidence-based decision-making domain. What appears to be occurring simultaneous to more standard forms of EBA is a rearticulation, or perhaps even couching, of ideological convictions in the authoritative language of scientific evidence for the sake of political expediency. Though many of our informants have begun to skeptically point to EBA's reductionist tendencies, they are also finding ways to continue using the power of science and objectivity to fulfill their enduring commitment to ethical and moral principles. Ideological debates and subjective values are thus not being eliminated but rather obfuscated as scientific and hence objective, and are reintroduced in ways that are more readily fungible with an evidence-based framework.

Such multi-layered appeals to objectivity demonstrate the extent to which SMI experts’ critical awareness and resistant activities are indeed constrained by the “technical infrastructures, administrative apparatuses [and] value regimes” of global forms (Collier and Ong 2005:11). Fostering such a heavy reliance on purportedly objective claims, rather than challenging the basis on which global-level decision-making takes place through explicitly value-based political arguments, may have negative consequences, as many of those contributing to these trends acknowledge.

Though the adoption of EBA aims to “save” the SMI as well as the women on whose behalf it advocates, it has contributed to making it more difficult to advance principled arguments about the importance of maternal health, unless these are articulated in terms of instrumental scientific, technical, or economic rationales. The technocratic priority-setting tools that are now dominating sustain a narrow cost-effectiveness focus not easily applied to the kinds of broader health system developments maternal health specialists say they are convinced are essential for reducing maternal mortality. By participating in EBA, maternal health advocates are also contributing to the unwelcome fragmentation of global health governance and national health systems that results from different professional community advocating for the uptake of their own set of issues and interventions. As a result of such competition, little attention is directed to cross-cutting issues central to the functioning of the overall health system or social and economic determinants of health (McCoy 2009). And even less attention is given to the political changes needed to address the health inequities that maternal mortality illustrates so clearly (Janes and Chuluundorj 2004; Spangler 2011).

Maternal health advocates’ experiences highlight that the pressure to participate in the numbers game, as well as the ambivalence with which players approach this game, emerge out of the messy and contradictory everyday life of global health politics. However, by pandering to the politics of global health, safe motherhood advocates may be extending the dominance of a technocratic approach that is at odds with the underlying political agenda they have been so keen to support since the SMI's inception.

## References

[b1] Adams V (2005). Saving Tibet? An Inquiry into Modernity, Lies, Truths, and Beliefs. Medical Anthropology.

[b2] Adams V, Metzl J, Kirkland A (2010). Against Global Health? Arbitrating Science, Non-science and Nonsense through Health. Against Health: How Health Became a New Morality.

[b3] Adams V, Novotny TE, Leslie H (2008). Global Health Diplomacy. Medical Anthropology.

[b4] Béhague DP, Storeng KT (2008). Collapsing the Vertical-Horizontal Divide: An Ethnographic Study of Evidence-based Policymaking in Maternal Health. American Journal of Public Health.

[b5] Béhague DP, Storeng KT (2013). Pragmatic Politics and Epistemological Diversity: The Contested and Authoritative Uses of Historical Evidence in the Safe Motherhood Initiative. Evidence & Policy.

[b6] Berry NS (2010). Unsafe Motherhood: Mayan Maternal Mortality and Subjectivity in Post-war Guatemala.

[b7] Biehl J, Petryna A (2013). When People Come First: Critical Studies in Global Health.

[b8] Birn A-E (2009). The Stages of International (Global) Health: Histories of Success or Successes of History?. Global Public Health.

[b9] Brown TM, Cueto M, Fee E (2006). The World Health Organization and the Transition from “International” to “Global” Public Health. American Journal of Public Health.

[b10] Buse K, Walt G (1997). An Unruly Mélange? Coordinating External Resources to the Health Sector: A Review. Social Science & Medicine.

[b11] Campbell O, Koblinsky M, Taylor P (1995). Off to a Rapid Start: Appraising Maternal Mortality and Services. International Journal of Gynaecology & Obstetrics.

[b12] Castro A, Singer M (2004). Unhealthy Health Policy: A Critical Anthropological Examination.

[b13] Chapman RR (2003). Endangering Safe Motherhood in Mozambique: Prenatal Care as Pregnancy Risk. Social Science & Medicine.

[b14] Collier SJ, Ong A, Collier SJ, Ong A (2005). Global Assemblages, Anthropological Problems. Global Assemblages: Technology, Politics, and Ethics as Anthropological Problems.

[b15] Countdown to 2015 (2009). http://www.countdown2015mnch.org.

[b16] Daston L (1992). Objectivity and the Escape from Perspective. Social Studies of Science.

[b17] Dobrow MJ, Goel V, Upshur REG (2004). Evidence-based Health Policy: Context and Utilisation. Social Science & Medicine.

[b18] Freedman LP (2003). Strategic Advocacy and Maternal Mortality: Moving Targets and the Millennium Development Goals. Gender and Development.

[b19] Gill K, Pande R, Malhotra A (2007). Women Deliver for Development. The Lancet.

[b20] Graham WJ, Campbell OM (1992). Maternal Health and the Measurement Trap. Social Science & Medicine.

[b21] Greenhalgh S (1996). The Social Construction of Population Science: An Intellectual, Institutional, and Political History of Twentieth-Century Demography. Comparative Studies in Society and History.

[b22] Greenhalgh T, Wieringa S (2011). Is It Time to Drop the “Knowledge Translation” Metaphor? A Critical Literature Review. Journal of the Royal Society of Medicine.

[b23] Hacking I (2007). British Academy Lecture 2006: Kinds of People, Moving Targets. Proceedings of the British Academy.

[b24] Haraway D (1988). Situated Knowledges: The Science Question in Feminism and the Privilege of Partial Perspective. Feminist Studies.

[b25] Hardon A (2005). Confronting the HIV/AIDS Epidemic in Sub-Saharan Africa: Policy versus Practice. International Social Science Journal.

[b26] Janes CR, Chuluundorj O (2004). Free Markets and Dead Mothers: The Social Ecology of Maternal Mortality in Post-Socialist Mongolia. Medical Anthropology Quarterly.

[b27] Janes CR, Corbett KK (2009). Anthropology and Global Health. Annual Review of Anthropology.

[b28] Justice J (1986). Policies, Plans, and People: Foreign Aid and Health Development. Volume 17.

[b29] Kapilashrami A, McPake B (2012). Transforming Governance or Reinforcing Hierarchies and Competition: Examining the Public and Hidden Transcripts of the Global Fund and HIV in India. Health Policy & Planning.

[b30] Keck ME, Sikkink K (1998). Activists beyond Borders: Advocacy Networks in International Politics.

[b31] Lakoff A (2010). Two Regimes of Global Health. Humanity: An International Journal of Human Rights, Humanitarianism and Development.

[b32] Lambert H (2006). Accounting for EBM: Notions of Evidence in Medicine. Social Science & Medicine.

[b33] Lambert H (2013). Plural Forms of Evidence in Public Health: Tolerating Epistemological and Methodological Diversity. Evidence & Policy: A Journal of Research, Debate and Practice.

[b34] Lancet The (2007). Women Deliver Press Conference and Press Release.

[b35] Mahler H (1987). The Safe Motherhood Initiative: A Call to Action. The Lancet.

[b36] McCoy D (2009). Global Health Initiatives and Country Health Systems. The Lancet.

[b37] McCoy D, Storeng KT, Filippi V, Ronsmans C, Osrin D, Borchert M, Campbell OM, Roques A, Wolfe R, Prost A, Hill Z (2010). Maternal, Neonatal and Child Health Interventions and Services: Moving from Knowledge of What Works to Systems that Deliver. Journal of International Health.

[b38] Mosse D (2011). Adventures in Aidland: The Anthropology of Professionals in International Development.

[b39] Nader L (1996). Naked Science: Anthropological Inquiry into Boundaries, Power and Knowledge.

[b40] Nichter M (2008). Global Health: Why Cultural Perceptions, Social Representations, and Biopolitics Matter.

[b41] Nichter M, Kendall C (1991). Beyond Child Survival: Anthropology and International Health in the 1990s. Medical Anthropology Quarterly.

[b42] Ong A (2007). Neoliberalism as a Mobile Technology. Transactions of the Institute of British Geographers.

[b43] Pfeiffer J, Nichter M (2008). What Can Critical Medical Anthropology Contribute to Global Health? A Health Systems Perspective. Medical Anthropology Quarterly.

[b44] Pigg SL, Davis-Floyd R, Sargent C (1997). Authority in Translation: Finding, Knowing, Naming and Training “Traditional Birth Attendants” in Nepal. Childbirth and Authoritative Knowledge: Cross-cultural Perspectives.

[b45] PMNCH (2011). Analysing Commitment to Advance the Global Strategy for Women's and Children's Health. The PMNCH 2011 Report.

[b46] Porter TM (1995). Trust in Numbers: The Pursuit of Objectivity in Science and Public Life.

[b47] Power M (1997). The Audit Society: Rituals of Verification.

[b48] Sabatier PA, Jenkins-Smith HC (1993). Policy Change and Learning: An Advocacy Coalition Approach.

[b49] Sai FT (1987). The Safe Motherhood Initiative: A Call for Action. IPPF Medical Bulletin.

[b50] Shiffman J (2007). Has Donor Prioritization of HIV/AIDS Displaced Aid for Other Health Issues?. Health Policy and Planning.

[b51] Shore C, Shore C, Wright S, Wright S (1997). Policy: A New Field of Anthropology. Anthropology of Policy: Critical Perspectives on Governance and Power.

[b52] Shore C, Wright S (1999). Audit Culture and Anthropology: Neo-liberalism in British Higher Education. The Journal of the Royal Anthropological Institute.

[b53] Spangler SA (2011). “To Open Oneself Is a Poor Woman's Trouble”: Embodied Inequality and Childbirth in South-Central Tanzania. Medical Anthropology Quarterly.

[b54] Starrs A (1987). Preventing the Tragedy of Maternal Deaths: Report of the Safe Motherhood Conference held in Nairobi, Kenya, February.

[b55] Storeng KT (2010). Safe Motherhood: The Making of a Global Health Initiative. Ph.D. Thesis.

[b56] Storeng KT, Mold A, Reubi D, Béhague DP (2013). Evidence-Based Advocacy and the Reconfiguration of Rights Language in Safe Motherhood Discourse. Health Rights in Global Context: Geneaologies and Anthropologies.

[b57] Strathern M (2000). Audit Cultures: Anthropological Studies in Accountability, Ethics, and the Academy.

[b58] Sundby J (1999). Are Women Disfavoured in the Estimation of Disability Adjusted Life Years and the Global Burden of Disease?. Scandinavian Journal of Public Health.

[b59] Wallack L, Dorfman L, Jernigan D, Themba-Nixon M (1993). Media Advocacy and Public Health: Power for Prevention.

[b60] Wedel JR, Feldman G (2005). Why An Anthropology of Public Policy?. Anthropology Today.

[b61] WHO (2004). Beyond the Numbers: Reviewing Maternal Deaths and Complications to Make Pregnancy Safer.

[b62] WHO and UNICEF (1978). Declaration of Alma-Ata. International Conference on Primary Health Care, Alma-Ata, USSR, 6–12 September 1978.

[b63] Women Deliver (2007). http://www.womendeliver.org/conferences/2007-conference/.

